# Anticancer therapeutic effect of ginsenosides through mediating reactive oxygen species

**DOI:** 10.3389/fphar.2023.1215020

**Published:** 2023-07-26

**Authors:** Xiaonan Li, Donghui Cao, Siming Sun, Yuehui Wang

**Affiliations:** ^1^ Department of Geriatrics, The First Hospital of Jilin University, Changchun, China; ^2^ Department of Clinical Research, The First Hospital of Jilin University, Changchun, China

**Keywords:** ginsenoside, cancer, reactive oxygen species, treatment, signaling pathways

## Abstract

Dysregulation of reactive oxygen species (ROS) production and ROS-regulated pathways in cancer cells leads to abnormal accumulation of reactive oxygen species, displaying a double-edged role in cancer progression, either supporting transformation/proliferation and stimulating tumorigenesis or inducing cell death. Cancer cells can accommodate reactive oxygen species by regulating them at levels that allow the activation of pro-cancer signaling pathways without inducing cell death via modulation of the antioxidant defense system. Therefore, targeting reactive oxygen species is a promising approach for cancer treatment. Ginsenosides, their derivatives, and related drug carriers are well-positioned to modulate multiple signaling pathways by regulating oxidative stress-mediated cellular and molecular targets to induce apoptosis; regulate cell cycle arrest and autophagy, invasion, and metastasis; and enhance the sensitivity of drug-resistant cells to chemotherapeutic agents of different cancers depending on the type, level, and source of reactive oxygen species, and the type and stage of the cancer. Our review focuses on the pro- and anticancer effects of reactive oxygen species, and summarizes the mechanisms and recent advances in different ginsenosides that bring about anticancer effects by targeting reactive oxygen species, providing new ideas for designing further anticancer studies or conducting more preclinical and clinical studies.

## 1 Introduction

Reactive oxygen species (ROS), as by-products of cellular respiration and aerobic metabolism, are a group of highly active oxygenated molecules that play a pivotal role in body function (Zheng et al., 2022). Redox reactions maintain ROS balance in the organism and function as signaling molecules to trigger cellular regulatory pathways. Extensive evidence suggests that ROS and abnormal redox reactions damage DNA, proteins, and lipids; thus, ROS are thought to be genomically damaging and cancer-promoting (Ismail et al., 2019). It has been well-established that almost all cancer cells exhibit variable types of oxidative stress and higher levels of ROS (Hayes et al., 2020). To adapt to high ROS levels and optimize their pro-cancer effects, carcinoma cells intelligently strengthen their antioxidant capacity to maximize their profitability during different stages (Reczek and Chandel, 2017; Maslah et al., 2020; Attanasio et al., 2021). However, different opinions have been raised that ROS in cancers act as double-edged swords. ROS not only help in cancer cell transformation/proliferation but also exert cytotoxicity, activate anticancer signaling, and promote oxidative stress-induced cancer cell death (Schieber and Chandel, 2014; Cheung et al., 2020; Ding et al., 2020; Zhang et al., 2020; Cruz-Gregorio et al., 2021; Kuo et al., 2022). This property makes these cells more susceptible to redox manipulation or altered ROS levels than normal cells (Gorrini et al., 2013; Reczek and Chandel, 2017). Hence, ROS modulation is a prospective approach for cancer treatment.

Ginseng (*Panax ginseng* C.A. Meyer), a perennial herb of the genus *Panax*, has a medicinal history of thousands of years and is widely used as a health food worldwide because of its medicinal properties (Kim, 2018). Ginseng, with a variety of pharmacological effects, including anticancer, antioxidant, anti-inflammatory, and other biological effects (Fu et al., 2018; Metwaly et al., 2019; Wang et al., 2021a), contains many chemical components, of which the most dominant are ginsenosides. Numerous studies have suggested that ginsenosides protect cells by preventing oxidative damage, thereby preventing the occurrence and progression of diseases (Kang et al., 2013; Ong et al., 2015). Possible mechanisms mentioned in previous studies include their ability to inhibit oxidative damage by inhibiting malondialdehyde (MDA) formation, reducing lipid peroxidation, and regulating the activity of antioxidant enzymes, such as superoxide dismutase (SOD), catalase (CAT), glutathione peroxidase (GPx), and other antioxidant factors (Kim et al., 2004). In addition, it has also been suggested that modulation of oxidative stress-related oxidative signaling pathways, such as the Keap1/Nrf2/ARE, PI3K/Akt, and Wnt/β-catenin, and nuclear factor-k-gene binding (NF-κB) signaling pathways, is also an important way in which ginsenosides exert antioxidant damage (Li et al., 2016; Chen et al., 2019; Hagar et al., 2019).

Given the heightened sensitivity to changes in ROS levels, a strategy to modulate ROS levels is likely to be valid for cancer therapy. Coincidentally, ginsenosides have a superior antioxidant capacity, and studies have also suggested that ginsenosides hold a central position in cancer therapy by modulating this target to control apoptosis and autophagy, stall cancer invasion and metastasis, regulate cell cycle arrest, and enhance the sensitivity of drug-resistant cells to chemotherapeutic agents; however, the specific mechanisms studied have not been fully elucidated (Zheng et al., 2016; Hong and Fan, 2019b; Han et al., 2020; Ping et al., 2020; Lu et al., 2022). Therefore, in this article, we focus on the pro- and anticancer effects of ROS and investigate the particular mechanisms and latest advances of different ginsenosides, including their derivatives and drug delivery, exerting anticancer effects by targeting ROS to represent new insights for further design of anticancer studies or conduct more preclinical and clinical studies.

## 2 Source of ROS and the modulation of ROS

**FIGURE 1:**
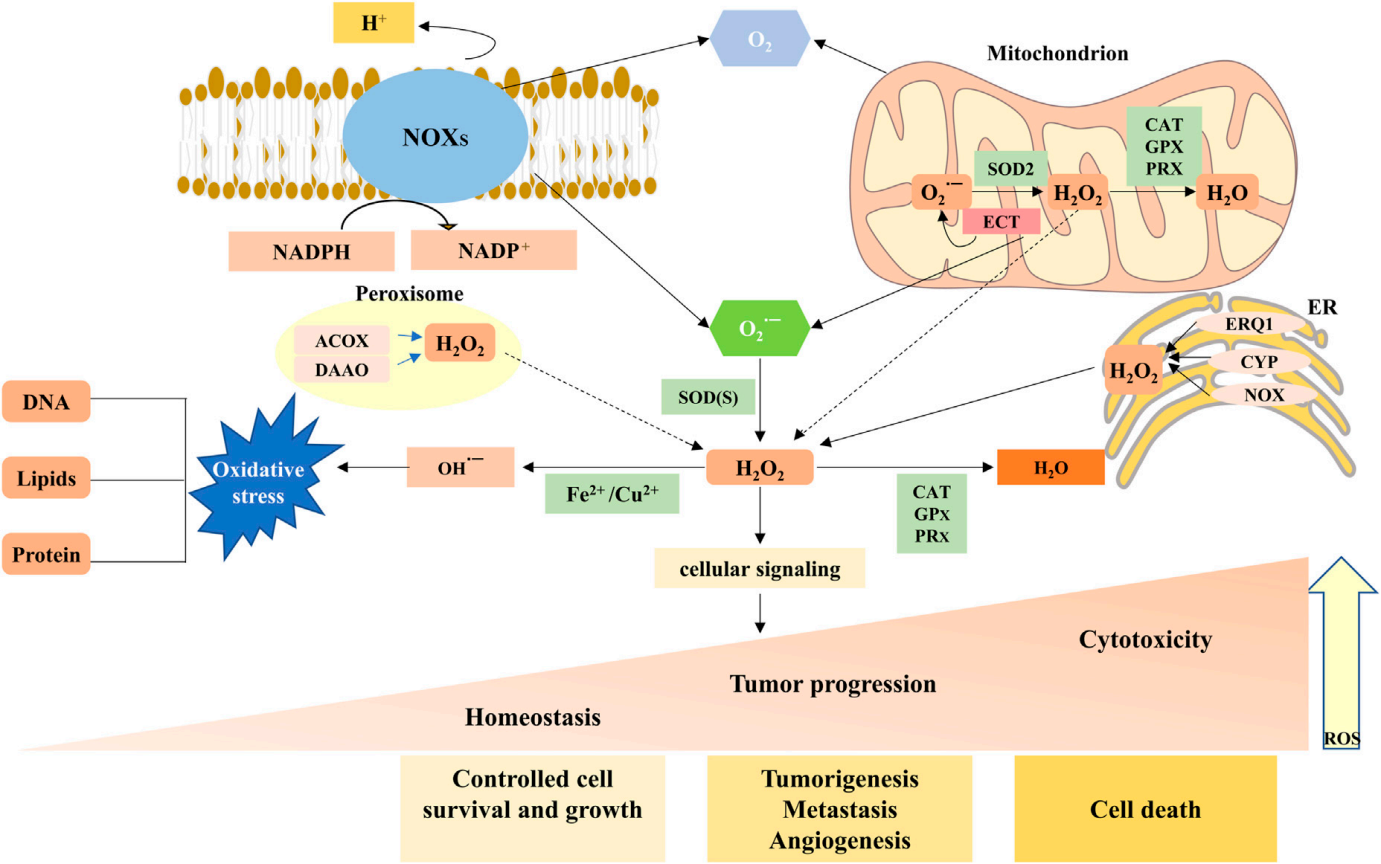
Main generation and modulation of ROS. Mitochondrial and membrane NADPH oxidases (NOXs) are the main sources of endogenous ROS production. The superoxide dismutase (SOD) enzymes transform superoxide radical anion (O_2_^·−^) into hydrogen peroxide (H_2_O_2_). H_2_O_2_ can undergo Fenton chemistry with Fe^2+^ to form a hydroxyl radical (OH^·−^), causing damage to DNA, proteins, and lipids. H_2_O_2_ can be reduced and converted to H_2_O by peroxiredoxins (PRXs), glutathione peroxidases (GPXs), and catalase (CAT). ROS, as a signaling molecule, impacts the development and progression of normal body and disease cells depending on its concentration.

**FIGURE 2:**
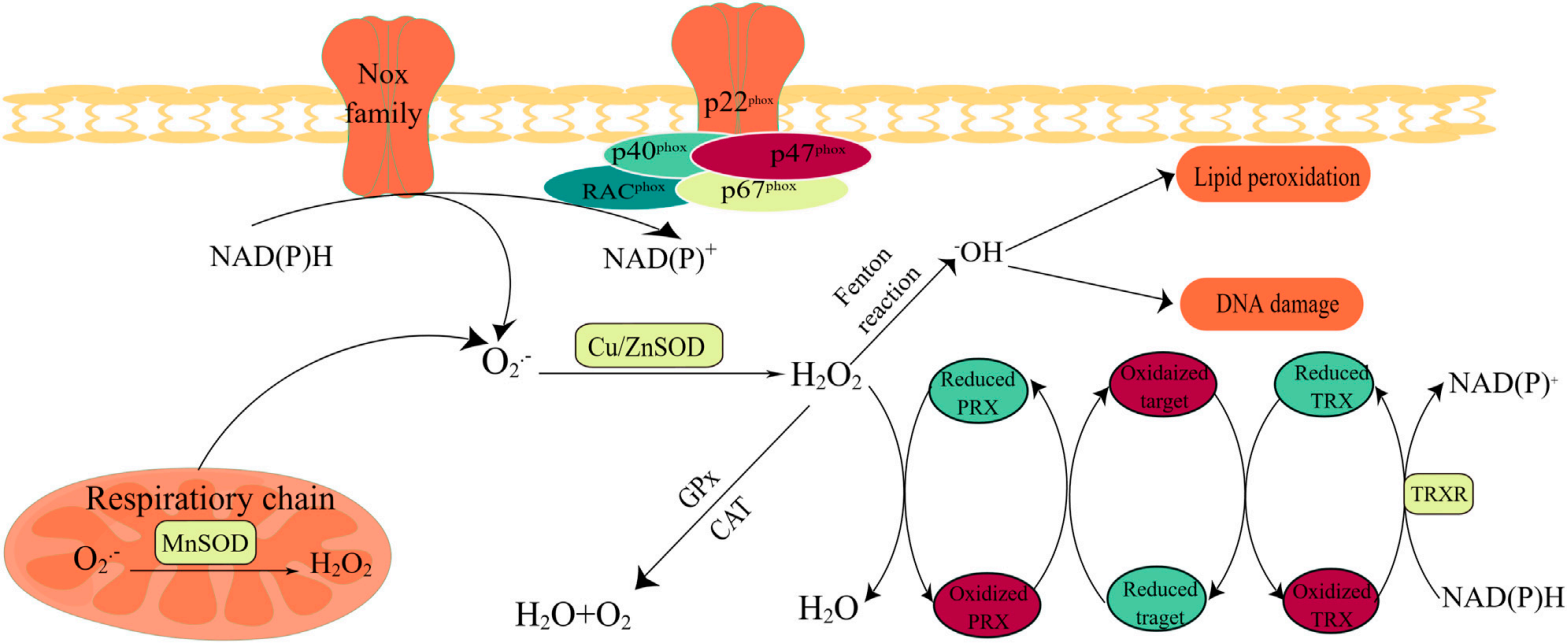
Oxidant and antioxidant enzymes. Activation of NOXs and the mitochondrial respiratory chain are the main sources of the superoxide radical anion (O_2_^·−^), and O_2_^·−^ conversion to H_2_O_2_ can be performed by (SOD. The activation of the NOX family requires the involvement of subunits such as Rac^phox^, p47^phox^, and p22^phox^; thus, the catalytic subunit can remove an electron from the cell membrane NADPH and transfer it to O_2_ to produce O_2_^·−^. CAT and/or GPX is modulated to maintain homeostasis in the body; when redox metals are present, increased concentrations of H_2_O_2_ produce hydroxyl radicals. Hydrogen peroxide oxidase (PRX) plays a critical role in hydrogen peroxide scavenging and redox regulation. Redox-regulated proteins can act as an extra redox relay base between PRX and thioredoxin (TRX).

ROS, which are single unpaired electrons present in free radicals, ions, or molecules, are a group of highly reactive chemicals that contain oxygen, including superoxide anions (O_2_^−^), hydrogen peroxide (H_2_O_2_), and hydroxyl radicals (OH^−^) (Martínez-Cayuela, 1995). Of these, H_2_O_2_ performs a critical role in signaling by selectively modifying and regulating the function of numerous proteins, whereas other forms of ROS are more likely to cause damage and toxicity (Cheung and Vousden, 2022). The primary cellular source of ROS is the mitochondria, which produce ROS during respiration as a natural by-product of electron transport chain (ETC) activity (Bertram et al., 2006) (Figure 1). Superoxide molecules are produced in complexes I and III and subsequently released into the intermembrane space and mitochondrial matrix (Han et al., 2001; Chen et al., 2003). Next, superoxide molecules in the mitochondrial outer membrane leak into the cytoplasm through the mitochondrial permeability transition pore (MPTP), whereas superoxide molecules in the mitochondrial matrix or cytosol are disproportionated to H_2_O_2_ by MnSOD and Cu/ZnSOD, respectively (Liou and Storz, 2010). In addition to the mitochondria, active nicotinamide adenine dinucleotide phosphate (NADPH) oxidases (NOXs) are another major source of ROS (Brown and Griendling, 2009; Bae et al., 2011). Normally, the NOX family is activated by Rac^phox^, p47^phox^, p22^phox^, p67^phox^, and 40^phox^ to allow the catalytic subunit to remove an electron from the cell membrane of NADPH and transfer it to O_2_ to produce O_2_^−^(Moloney and Cotter, 2018) (Figure 1 and Figure 2). Moreover, there is striking evidence that ROS produced by NOXs as signaling molecules can regulate cell behaviors such as cell proliferation, differentiation, and apoptosis. The aberrant expression of NOXs can induce various diseases, including cancer (Bonner and Arbiser, 2012; Spencer and Engelhardt, 2014).

Mitochondria and NOXs, the two main sources of ROS, mediate ROS-dependent signaling, an effect that can be achieved by spatial localization to oxidant receptors. H_2_O_2_ has strong diffusion properties and is brought into the cytoplasm exclusively by aquaporins (aquaporins 3 and 8) as secondary messengers that regulate multiple signaling pathways (Wang et al., 2021b). However, when accompanied by excessive ROS levels, H_2_O_2_ can move away from the site of production, inducing oxidative damage and cell death. Therefore, whether ROS are a poison or cure depends largely on the local concentration and type of ROS, as well as the abundance of antioxidants (Chandel and Tuveson, 2014).

Other sources of ROS come from misfolding of proteins through endoplasmic reticulum (ER) stress (Murphy, 2013); cytochrome P450 (CYP) (Kim et al., 2020); Fenton chemistry that involves transition metal ions (Halcrow et al., 2021) and external stimuli, such as epidermal growth factor (EGF), cancer necrosis factor-α (TNF-α), and interleukin-1β (IL-1β) irradiation; and hypoxia (Ilatovskaya et al., 2013; Clauzure et al., 2014; Roberge et al., 2014; Cheung and Vousden, 2022).

## 3 Types of antioxidants

Unsurprisingly, healthy cells have evolved to overcome the damaging effects of ROS, including balanced ROS production, adequate antioxidant activity, and cellular repair, leading to the maintenance of ROS at an equilibrium concentration. At low-to-moderate levels, ROS can act as cell signaling messengers involved in regulating a variety of cellular functions; however, high levels of ROS can cause DNA damage, lipid peroxidation, and protein oxidation, causing cell damage (Bae et al., 2011; Chio and Tuveson, 2017; Reczek and Chandel, 2017). Under normal physiological conditions, contributing to a collectively powerful antioxidant system, intracellular ROS levels are steadily kept in balance to ensure that the ROS signaling process is maintained smoothly while avoiding oxidative damage. The generation and modulation of ROS, and the functions of oxidant and antioxidant enzymes are shown in Figure 1 and Figure 2, respectively.

SODs, categorized as cell membrane SOD1, mitochondrial SOD2, and extracellular SOD3, are vital enzymes in the antioxidant enzyme defense system, particularly superoxide anion radicals (which catalyze the conversion of O_2_^−^ to H_2_O_2_ and O_2_) (Areti et al., 2014; Sheng et al., 2014).SODs may also attenuate NOX-dependent redox reactions by regulating diffuse H_2_O_2_ signaling and signaling-related activation of receptor tyrosine kinases and G-protein-coupled receptors (Parascandolo and Laukkanen, 2018). Despite being termed carcinogens, some SODs can also be upregulated during carcinogenesis (Hayes et al., 2020). To maximize the benefits of H_2_O_2_ levels for the body, several other antioxidants are involved in the intracellular conversion of H_2_O_2_ to H_2_O, including peroxidases (PRXs), glutathione peroxidases (GPXs), and CAT (Brigelius-Flohé and Maiorino, 2013). PRXs, which have a high-affinity binding site for H_2_O_2_ and are abundantly expressed in subcellular compartments, are considered to be ideal H_2_O_2_ scavengers (Wang et al., 2021b). GPXs convert H_2_O_2_ to H_2_O by oxidizing reduced glutathione (GSH) to glutathione disulfide (GSSG), and glutathione reductase (GR) uses NADPH as an electron donor to convert oxidized GSSG to GSH. Distinguishing from PRXs and GPXs, CAT participates in two different antioxidant reactions depending on the H_2_O_2_ concentration (Sepasi Tehrani and Moosavi-Movahedi, 2018). At high H_2_O_2_ levels, CAT exhibits catalytic activity, transforming H_2_O_2_ into H_2_O_2_ and O_2_. However, low H_2_O_2_ levels display peroxidative activity, decreasing one H_2_O_2_ molecule to two H_2_O molecules, by depleting two reducing equivalents from non-NADPH hydrogen donors, such as alcohols, phenols, hormones, and metals (Wang et al., 2021b).

The main members of the nonenzymatic antioxidant system include water-soluble small molecules, fat-soluble antioxidants, protein-based antioxidants, and trace elements, which form the body’s second line of defense (Birben et al., 2012; Gamcsik et al., 2012; Forman and Zhang, 2021; Jelic et al., 2021). Reduced GSH and NADPH levels have been the focus of many studies. GSH directly or indirectly reacts with oxidizing substances and is oxidized to GSSG (Gaucher et al., 2018). Radicals and ROS are directly quenched by GSH. Under enzymatic action, GSH functions as a cosubstrate for GPX, reducing H_2_O_2_ and lipid peroxide to H_2_O and lipid alcohols (lipid-OOH) (Niu et al., 2021). NADPH, an indispensable product of multiple intracellular metabolic pathways, is a highly desirable electron donor that restores oxidized GSH and thioredoxins (TXNs) produced by the reduction of GPXs and PRXs to a reduced state (Sies and Jones, 2020). As an electron carrier, NADPH is not only a producer (incompletely reduced) but also a scavenger (completely reduced) of ROS. Paradoxically, inadequate NADPH leads to ROS accumulation, and excessive NADPH leads to reductive stress, which is utilized by NOXs to produce ROS. Therefore, only if the NADP+/NADPH level remains in an equilibrium state can NADPH perform its antioxidant defensive roles (Wang et al., 2021b).

Many transcription factors, including nuclear factor E2-related factor (Nrf2), oncoprotein p53, activator protein 1 (AP-1), HIF-1a, nuclear factor κB (NF-κB), and forkhead box O class (FOXO), can be activated by ROS and regulate the redox state of cells (Marinho et al., 2014). (Marinho et al., 2014).

## 4 Function of ROS in cancer

**FIGURE 3:**
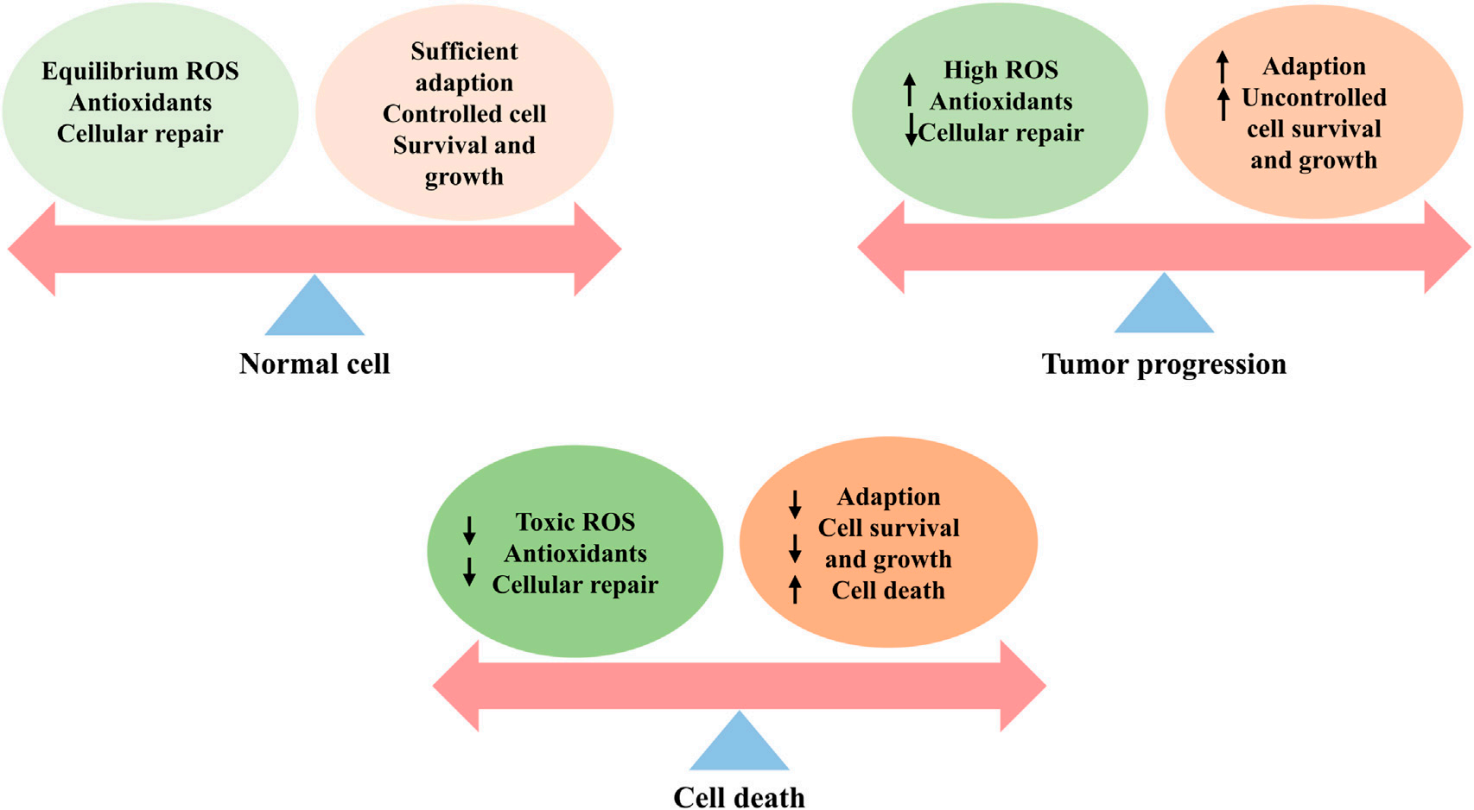
ROS effects on cells, including the normal cells, tumor progression, and cell death. Healthy cells have a well-balanced production of ROS, adequate antioxidant activity, and cellular repair, resulting in appropriate concentrations of ROS that limit cell survival and proliferation. Increased levels of ROS can cause cellular damage, yet tumor cells express the enhanced antioxidant activity and maintain pro-tumor signaling through adequate adaptation to conditions including hypoxia and through metabolic readjustment. However, ROS levels increase to toxic concentrations, and oxidative stress leads to irreparable damage to cells, inadequate adaptation, and, ultimately, tumor cell death.

**FIGURE 4:**
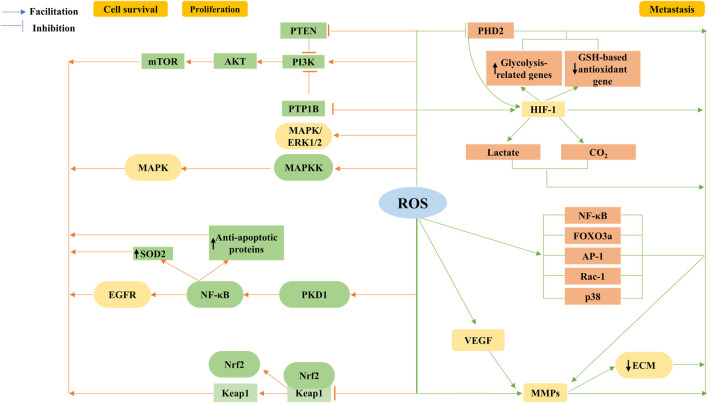
Tumorigenic role of ROS. ROS are a second messenger involved in growth factor activation via PI3K/Akt/mTOR, and mitogen-activated protein kinase (MAPK)/extracellular regulated protein kinase (ERK) proliferation survival-related signaling cascades, regulation of the nuclear factor κB (NF-κB) activation pathway, and mutations in nuclear factor E2-related factor (Nrf2) are involved. ROS enhance cancer cell migration and invasion by stabilizing hypoxia-inducible factor-1α (HIF-1α) subunits through inhibition of the prolyl hydroxylase domain protein (PHD2) in the hypoxia signaling pathway. HIF-1 upregulates lactate dehydrogenase and pyruvate dehydrogenase kinase 1, downregulates glutathione (GSH)-based antioxidant gene expression, and decreases mitochondrial ROS production, leading to increased lactate and carbon dioxide formation, all of which facilitate extracellular matrix degradation and cell invasion. The coordinated signaling cascade of HIF-1α completes growth factor-mediated angiogenesis, such as vascular endothelial growth factor (VEGF), while ROS also accelerate angiogenesis and promote metastasis by stimulating matrix metalloproteinase (MMP)-dependent extracellular matrix (ECM) protein degradation; in addition, ROS trigger transcription factors NF-κB and forkhead box O3 (FOXO3a) and Ras-related C3 botulinum toxin substrate 1 (Rac-1), activator protein-1 (AP-1), or p38 signaling pathways to alter MMP expression, promoting tumor progression.

**FIGURE 5:**
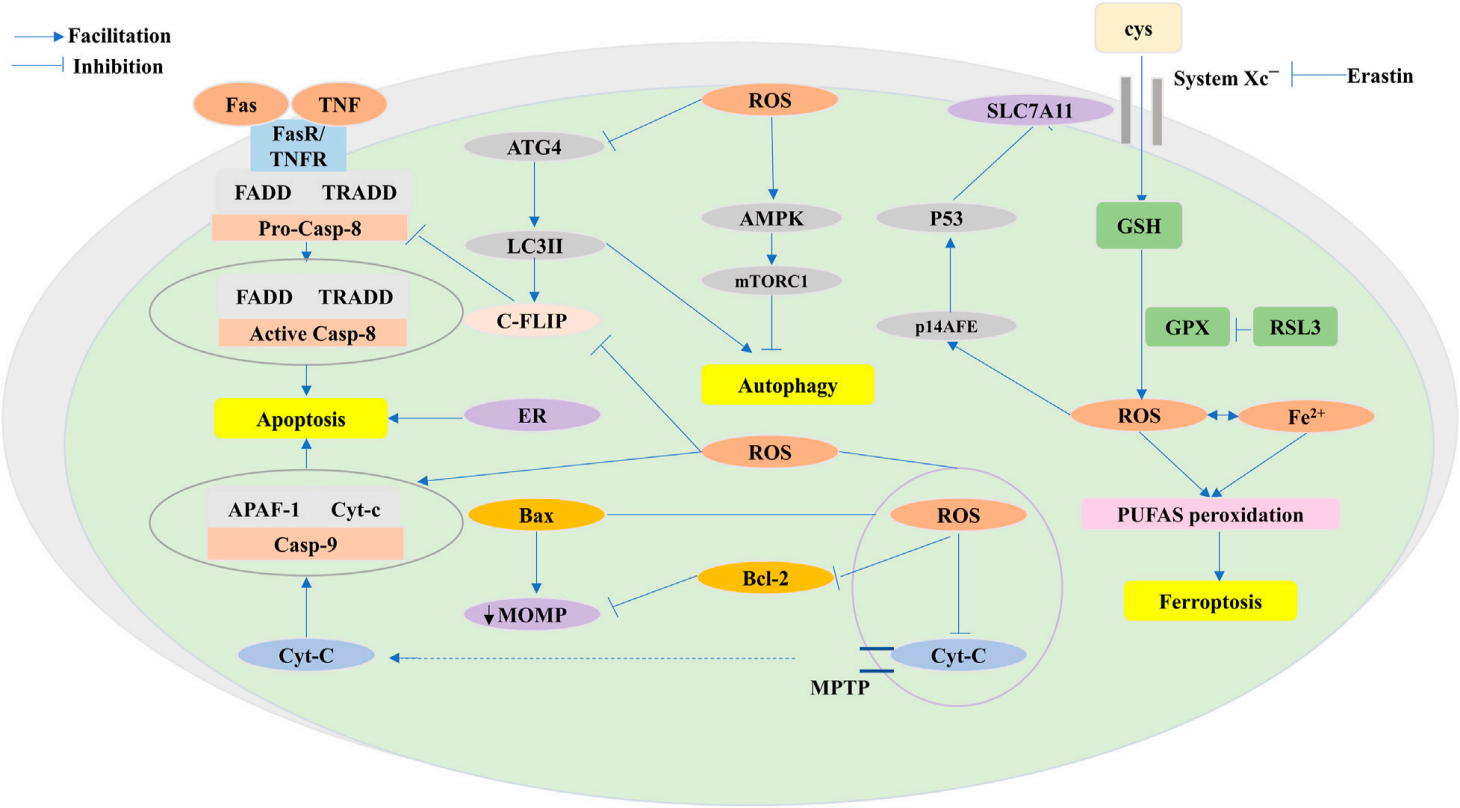
ROS functions of antitumor effects via programmed cell death (PCD) consisting largely of apoptosis, autophagy, and ferroptosis. ROS exert pressure on the open mitochondrial permeability transition pore (MPTP), leading to a decreased linear mitochondrial membrane potential (MMP), allowing cytochrome C (Cyt-c) to be released into the cytoplasm and form an apoptotic complex with apoptotic protease activating factor-1 (APAF-1) and procaspase-9 while triggering a caspase-9 signaling cascade that ultimately triggers apoptosis. ROS-dependent proteasomal degradation of cellular FADD-like IL-1β-converting enzyme (FLICE)-inhibitory protein (c-FLIP) enhances the exogenous apoptotic pathway, which is triggered by the splicing of death-inducing ligands with their cognate receptors (FasR). ROS-triggered endoplasmic reticulum (ER) stress is indispensable in apoptosis. ROS exert anticancer effects through autophagy. ROS-dependent inactivation of autophagy-related gene 4 (Atg4) leads to an increase in microtubule-associated protein 1 light chain 3 (LC3)-associated autophagosomes and induces autophagy. mTORC1, a negative regulator of autophagy, is activated by adenosine 5′-monophosphate (AMP)-activated protein kinase (AMPK) and inhibits autophagy. Ferroptosis is caused by ROS-induced iron-dependent lipid peroxidation of PCD. Fenton chemistry increases lipoxygenase activity/ROS production. Erastin impairs the GSH-dependent glutathione peroxidase (GPX) antioxidant system through the mediation of the cystine/glutamate reverse transporter protein (XC^−^ system). Increasing ROS causes changes in the permeability of the outer mitochondrial membrane, and (1S,3R)-RSL3 (RSL3) causes changes in the outer mitochondrial membrane by blocking GPX that stimulates ferroptosis. The P14 alternate reading frame (p14ARF) is triggered by ROS, activates P53 to downregulate recombinant solute carrier family 7, member 11 (SLC7A11), and affects ferroptosis.

The function of ROS in cancer remains unknown and warrants in-depth exploration. Sustained exposure to high ROS levels can be detrimental to DNA, causing oncogene activation and cancer suppressor gene inactivation, mediating signaling events, and promoting carcinogenesis, progression, and metastasis (Chio and Tuveson, 2017; Rose Li et al., 2020; Aboelella et al., 2021). Unlike normal cells that die from prolonged exposure to these conditions, cancer cells strategically activate antioxidant systems and metabolic changes, including the elevated activity of antioxidants such as SOD2/MnSOD or inactivation of scavenging enzymes such as PRX1 stabilization of HIF, and activation of AMPK, to strengthen the generation of NADPH and GSH and the maintenance of redox homeostasis to thrive in harsh cancers (Jeon et al., 2012; Reczek and Chandel, 2017), thereby promoting carcinogenesis and progression (Chandel and Tuveson, 2014; Sies and Jones, 2020). However, when the accumulation of ROS crosses a certain threshold, their carcinogenic effects on proliferation and invasion are eliminated and transformed into antitumor effects via an induced regulated cell death (RCD) program that consists largely of apoptosis, autophagy, and ferroptosis (Wang et al., 2021b). Surprisingly, oxidative stress is a significant obstacle in the metastatic spread of cancer (Hayes et al., 2020). The role played by ROS in cancer, whether spear or shield, may depend on the genetic background of the cancer, the type of ROS involved, and the level and duration of ROS exposure. Figures 3–5 show the effects of ROS on normal cells, tumor progression, and cell death.

### 4.1 Cancerogenic role of ROS

#### 4.1.1 Proliferation and cell survival

The ROS-dependent stimulation of cell survival and proliferation has been extensively studied. The most compelling accomplishment is the confirmation that ROS are secondary messengers involved in growth factor activation via the PI3K/Akt/mTOR and MAPK/extracellular regulated protein kinase (ERK) proliferation survival-related signaling cascades (Moloney and Cotter, 2018). Moreover, regulation of the NF-κB activation pathway and mutations in transcription factors and oncogenes, including Nrf2 and p53, are also involved.

The Akt pathway, through the phosphorylation and inactivation of its target proteins, consists of proapoptotic Bad, Bax, and Foxo transcription factors, which are responsible for their role in cell survival (Limaye et al., 2005; Moloney and Cotter, 2018). Numerous studies have shown that the ROS-induced PI3K/Akt survival pathway is activated in most cancer types. Phosphatases, such as PTEN and PTP1B, contain cysteine active sites that act as negative regulators of the PI3K signaling pathway and are oncogenic agents that are inactivated by increased H_2_O_2_ oxidation, thereby affecting cell survival. Considering the importance of PI3K/Akt in the mitogenic signaling cascade, excessive activation of this pathway by the destructive oxidation upstream of PTEN/PTP1B is a hallmark of malignancy (Pérez-Ramírez et al., 2015). Consistent with this finding, we found that PTEN was inactivated and the ROS-induced PI3K signaling pathway was activated in various cancers. NOXs in neuroblastoma lead to the inactivation of the oncogene PTEN and activation of the PI3K/Akt signaling pathway in an ROS-dependent manner (Calvo-Ochoa et al., 2017). Similarly, in breast cancer cells, the accumulation of transient H_2_O_2_ by CXCL12-activated NOX2 leads to the oxidation of PTEN and PTP1B, regulating the activation of the PI3K/Akt signaling pathway and leading to the maintenance of cell cycle protein D expression, which, in turn, stimulates cell proliferation (Deng et al., 2018).

Activation of the MAPK/ERK 1/2 pathway, as a function of increased cell proliferation, can be associated with the stimulation of growth factors and K-Ras (Khavari and Rinn, 2007). Mitochondrial ROS regulate the K-Ras-induced anchorage-independent growth of lung cancer cells through the MAPK/ERK pathway (Weinberg et al., 2010). The proliferative actions of ovarian cancer cells in the presence of high levels of endogenous ROS are facilitated by sustained ubiquitination and inactivation of endogenous mitogen-activated protein kinase phosphatase 3 (MKP3) and increased ERK1/2 activity (Chan et al., 2008). Identical results were obtained when breast cancer cells were treated with ROS scavengers/inhibitors targeting ERK1/2 or its upstream kinase MAPKK (Zhou et al., 2008). In addition to initiating its upstream signaling pathway, the MAPK phosphoenzyme is directly oxidized and inactivated, sustaining JNK activation via the reversible oxidation of cysteine to sulfenic acid at the catalytic site and the inhibition of JNK-inactivating phosphatase (Kamata et al., 2005; Son et al., 2011). As is well known, ERK1/2 not only plays a proliferative role but also functions in the survival, anchorage-dependent growth, and motility of a variety of cancer cells (Liou and Storz, 2010; Moloney and Cotter, 2018).

ROS contribute to cancer cell survival and proliferation by activating NF-κB and Nrf2. Studies identified that the formation of pancreatic precancerous lesions was attributed to K-Ras-derived mtROS activating NF-κB via PKD1 to upregulate proliferative EGFR signaling (Liou et al., 2016). Increased mitochondrial ROS levels would also induce the upregulation of antioxidant proteins, such as MnSOD, and antiapoptotic proteins through the aforementioned pathways (Storz et al., 2005), thus helping in cancer survival and proliferation. Studies have also shown that the occurrence of various cancers is strongly correlated with mutations in Nrf2 (Rojo de la Vega et al., 2018). These findings suggest that the upregulation of Nrf2, either through increased Nrf2 mRNA production by oncogenic gene transcription (DeNicola et al., 2011) or the inhibition of its blocker KEAP1 upregulation by ROS (Suzuki et al., 2019), promotes preneoplastic nodules in the liver (Zavattari et al., 2015). Furthermore, differential activation of Nrf2, caused by the previously mentioned factors, is linked to cancer recurrence and unfavorable prognosis (Wu et al., 2019).

#### 4.1.2 Metastasis

Cancer metastasis is a complex multistep process, being one of the grounds for poor patient prognosis and treatment discouragement. The complex regulatory effects are reinforced by the fact that ROS function in cancer metastasis, allowing the regulation of key steps in cancer-increased migration, invasive potential, regulation of metabolism, epithelial–mesenchymal transition (EMT), and neovascularization (Liou and Storz, 2010).

The modulation of hypoxia contributes to the development of a malignant phenotype and aggressive cancer progression. PPP-dependent NADPH output is compromised by low glucose availability; subsequently, carcinoma cells accommodate glucose deprivation and protect against H_2_O_2_-induced apoptosis through the Warburg effect. Furthermore, the AMPK signaling pathway is activated to facilitate NADPH production and prevent anabolic processes that require NADPH depletion to optimize ROS yields, which significantly promotes the emergence of an aggressive phenotype (Aykin-Burns et al., 2009; Jeon et al., 2012; Ye et al., 2014; Vander Heiden and DeBerardinis, 2017).

The hypoxia-inducible factor-1 (HIF-1), composed of two subunits, HIF-1α and HIF-1β, potentially mediates cancer angiogenesis, metabolism, and metastasis, enhancing cancer survival and progression (Harris, 2002; Al Tameemi et al., 2019). ROS stabilize oxygen-sensitive HIF-α subunits by inhibiting PHD2 in the hypoxia signaling pathway (Bell et al., 2007), thereby enhancing cancer cell migration and invasion. Similarly, interest has been expressed in the increased expression of vascular endothelial growth factor (VEGF) genes and the activation of HIF-1α associated with metastatic disease (Semenza, 2012). The stability and activation of HIF-α through ROS-dependent mechanisms are strongly correlated with poor prognosis, increased cancer incidence (Horak et al., 2010), and aggressiveness in certain cancer cells. Furthermore, HIF-1 is responsible for the upregulation of glycolysis-related genes, such as lactate dehydrogenase and pyruvate dehydrogenase kinase 1, downregulation of GSH-based antioxidant gene expression (Lu et al., 2015; Stegen et al., 2016), and reduction of mitochondrial ROS production. It is equally intriguing to note that HIF-1 elicits increased lactate and carbon dioxide formation, and prevents intracellular acidification (Pouysségur et al., 2006), in both cases favoring extracellular matrix degradation and cell invasion (Rofstad et al., 2006).

EMT is the initial event in cancer cell metastasis. ROS promote metastasis by accelerating HIF-dependent angiogenesis through the stimulation of matrix metalloproteinase (MMP)-dependent ECM protein degradation (Chatterjee and Chatterjee, 2020; Aboelella et al., 2021). Another study also showed that ROS trigger the transcription factors NF-κB and FOXO3a and Rac-1, AP-1 (activator protein 1), or the p38 signaling pathway, to alter the expression of MMPs (Westermarck and Kähäri, 1999). It has also been reported that cancer cells induce the secretion of MMPs, such as MMP-1, via the upregulation of ROS to enhance vascular growth in the cancer microenvironment (Wartenberg et al., 2003). Angiogenesis, mediated by growth factors such as VEGF, can be accomplished through a coordinated signal transduction cascade by HIF-1α (Choudhry and Harris, 2018).

### 4.2 Anticancer role of ROS

#### 4.2.1 Anticancer effect of ROS through regulation of apoptosis

ROS have been proven to be excellent signaling molecules in the apoptotic process that directly induce cellular damage and activate caspase family protein-dependent activation of endogenous mitochondrial pathways and exogenous death receptor pathways (Redza-Dutordoir and Averill-Bates, 2016). ROS generate pressure against the open MPTP, causing a reduction in the mitochondrial transmembrane potential, thus contributing to the loss of membrane permeability and allowing the release of proapoptotic factors such as cytochrome c (Cyt-c) into the cytoplasm. Subsequently, once Cyt-c is released into the cytoplasm, its interaction with apoptosis protease-activating factor 1 (APAF-1) and pro-caspase-9 forms an apoptotic complex, simultaneously triggering a caspase-9 signaling cascade that breaks DNA and ultimately triggers apoptosis (Simon et al., 2000; Redza-Dutordoir and Averill-Bates, 2016; Moloney and Cotter, 2018). Furthermore, damaged mitochondria enable the production of more ROS, paradoxically accelerating apoptosis. In addition, ROS can modulate related signaling pathways and consequently cause apoptosis. Studies suggest that JNK, a subclass of the MAPK signaling pathway augmented by ROS, can respond by catalyzing the phosphorylation and downregulation of antiapoptotic proteins (Cadenas, 2004), increasing the expression of Bax, creating Bax homodimers, and ultimately destroying mitochondrial membrane integrity (Zhang et al., 2008). P38, another subclass of the MAPK signaling pathway, enthusiastically embraces apoptotic signaling enhanced by ROS production, and coincidentally, both of these can promote cell death through the involvement of apoptotic signal-regulated kinase 1 (Ask-1) (Saitoh et al., 1998; Liou and Storz, 2010). Moreover, it remains to be determined whether the ROS-mediated activity of the JNK and p38 signaling pathways is responsible for cell cycle arrest and inhibiting cancer cell growth and division (Wagner and Nebreda, 2009). Nevertheless, several other signaling proteins like P53 are also involved in ROS-induced apoptosis, and ROS above threshold levels initiate P53-induced genes, resulting in impaired mitochondrial function and triggering apoptotic effects (Safdar et al., 2016).

The extrinsic apoptotic pathway is triggered by the splicing of death-inducing ligands such as the Fas ligand (FasL) with their cognate receptors (FasR). After binding to the two receptors, the apoptotic message is transmitted to the cell interior, activating the linked death-inducing signaling complex (DISC), whose members include DD, DED, FADD (TRADD), and pro-caspase-8. Caspase-8 initiates a downstream caspase cascade that actively drives apoptosis. Cellular Fas-associated death domain-like interleukin 1β-converting enzyme inhibitor protein (c-FLIP) is a death receptor-mediated antiapoptotic factor. It has been shown that under conditions of threonine 166 phosphorylation and lysine 167 ubiquitination, the ROS-dependent degradation of the proteasome of c-FLIP reinforces the exogenous apoptotic pathway (Wilkie-Grantham et al., 2013). Additionally, in the case of N-acetylcysteine (NAC) pretreatment, the c-FLIP protein was efficiently stabilized and accelerated the initiation of apoptosis, confirming the role of ROS as an apoptosis-inducing factor (Moloney and Cotter, 2018). Moreover, ER stress and the regulation of energy metabolism triggered by ROS are indispensable for apoptosis.

#### 4.2.2 Anticancer effect of ROS through regulation of autophagy

Rather than acting as a mechanism for normal cell self-protection and survival, autophagy suppresses cancer and is referred to as type II programmed cell death. Ample evidence suggests that autophagy is directly mediated in malignancies and controlled by ROS levels (Poillet-Perez et al., 2015). The H_2_O_2_-dependent inactivation of autophagy-associated gene 4 (ATG4) leads to an increase in LC3-associated autophagosomes (Perillo et al., 2020), whereas oxidative stress in H_2_O_2_ and 2-ME-treated cancer cells also leads to autophagy-induced death (Chen et al., 2008). mTORC1, a negatively regulated regulator of autophagy (Moloney and Cotter, 2018), is suppressed by AMPK activation and contributes to autophagy induction. Autophagy in human bronchial epithelial cells is prevented by arsenic-induced ROS production. Notably, phosphorylation of AMPKK due to oxidative stress and the consequent alteration of the AMPK pathway further cause an increase in ROS and, eventually, apoptosis (Poillet-Perez et al., 2015). Last but not least, transcription factors, such as NF-κB, equally regulate autophagy by controlling the expression of genes that predominantly induce ROS-related autophagy in cancer, consequently regulating cell death (Boyer-Guittaut et al., 2014).

#### 4.2.3 Anticancer effect of ROS through regulation of ferroptosis

Ferroptosis is regulated by cell death (RCD), evoked by ROS-induced iron-dependent lipid peroxidation (Dixon et al., 2012). Peroxidized polyunsaturated fatty acids (PUFAs), triggered by oxidative stress involving excessive iron levels and insufficient GSH, are the underlying features of ferroptosis (Stockwell et al., 2017). There is ample evidence that intracellular iron, rendering a free state with redox activity, occurs through Fenton chemistry to increase lipoxygenase activity and ROS production (Stockwell et al., 2017). In contrast, membrane phospholipid-PUFAs are oxidized, which shows tremendous potential to shift membrane pores and integrity (Dixon and Stockwell, 2019). Studies have suggested that GPX4 utilizes GSH to reduce lipid hydroperoxides, thereby limiting ferroptosis (Kuang et al., 2020). In recent years, a mounting stream of data have emerged demonstrating that ferroptosis, specifically mediated by small molecules, exerts dramatic suppressive effects on cancer growth. Erastin, a synthetic small-molecule drug inhibiting cystine uptake, lends itself to an impairment of the GSH-dependent GPX antioxidant system via cystine/glutamate reverse transporter protein (system XC^−^) mediation, allowing for higher ROS levels and altered permeability of the outer mitochondrial membrane, causing cell death through ferroptosis in cancer cells, thus bearing mutant RAS (Yagoda et al., 2007; Dixon et al., 2012). The other agent, RAS-selective lethal compound 3 (RSL3), reportedly stimulates ferroptosis by blocking GPX, rather than the XC system (Imai et al., 2017). In addition, ROS trigger p14ARF (a cancer suppressor), which subsequently reactivates P53 and inhibits Nrf2 to activate ferroptosis and allow the downregulation of SLC7A11 and xCT activity (Chen et al., 2017). Numerous studies have confirmed that ferroptosis curtails cancer cell progression by eliminating these factors.

#### 4.2.4 Metastatic impairment

Cancer metastasis is inhibited by ROS overload (Piskounova et al., 2015). Remarkably, cancer cells can escape cell death during metastasis through metabolic alterations that are mediated by modulating the level of antioxidant capacity (Reczek and Chandel, 2017). Jiang et al. suggested that human melanoma cells promote metastasis by relying on NADPH-generating enzymes that rely on folate-mediated metabolism of single carbon units (Piskounova et al., 2015). Somewhat more interesting is the discovery that blood itself likely functioned as a prooxidant environment, provoking oxidative stress and hindering metastasis (Nieto et al., 2016).

## 5 Mechanism of ginsenoside inhibition of cancer development through the regulation of ROS levels

### 5.1 Overview of ginsenosides

**FIGURE 6:**
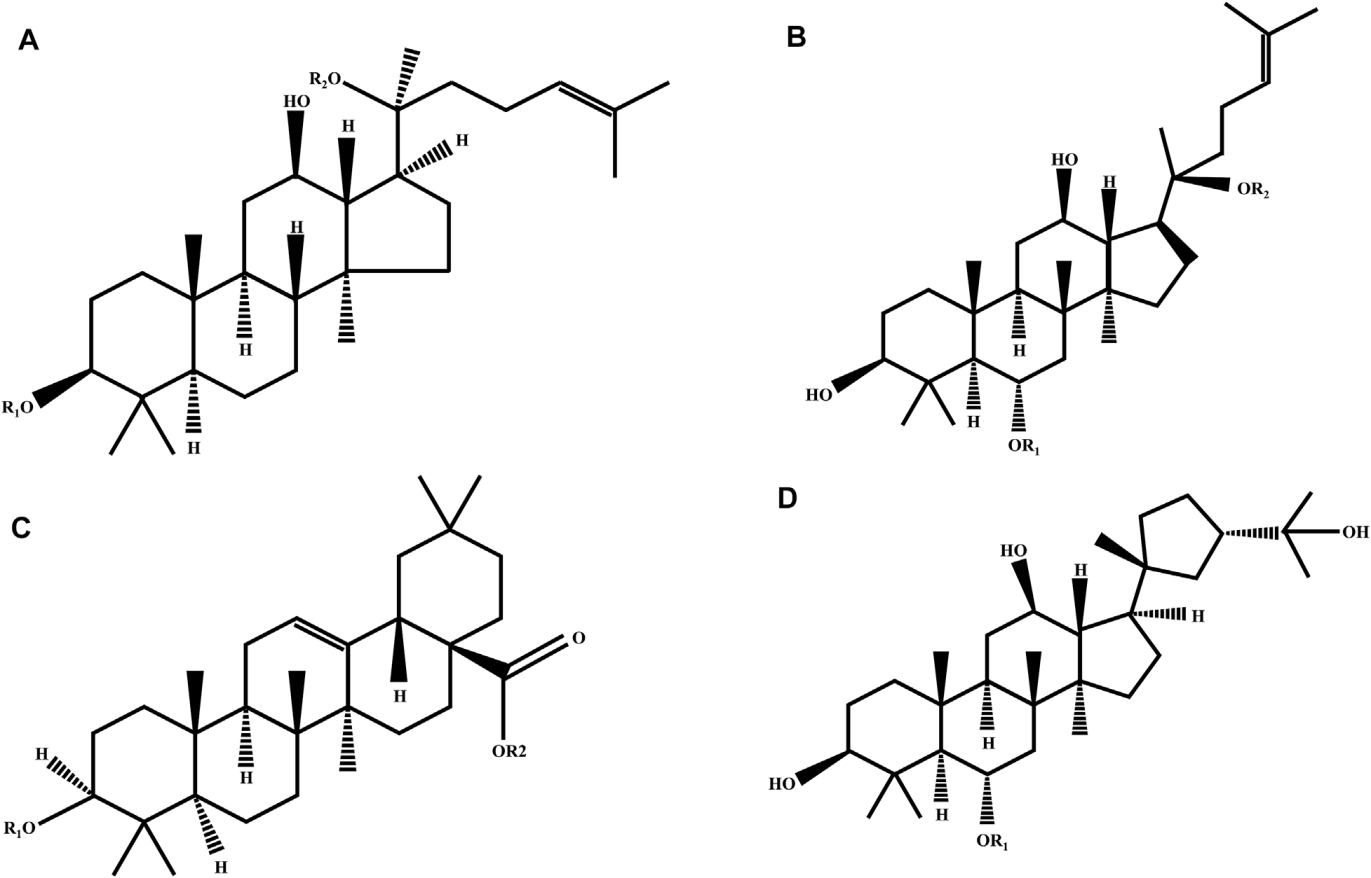
Chemical structure of four ginsenoside types. **(A)** Protopanaxadiol (PPD); **(B)** protopanaxatriol (PPD); **(C)** oleanolic acid; and **(D)** ocotillol ginsenosides.

Ginsenosides, the primary active components of ginseng, originate from the various pharmacological and biological effects of ginseng, affecting oxidative stress, metabolism, immunity, and the central nervous system, particularly when used in cancer therapy (Guo et al., 2019). Depending on the steroid backbone, hydroxyl groups attached, or number of sugar moieties, ginsenosides can be categorized into four types: protopanaxadiol-type (PPD), protopanaxatriol-type (PPT), oleanolic acid-type, and ocotillol-type. PPD-type ginsenosides include ginsenosides Rb1, Rb2, Rb3, Rc, Rd, Rg3, Ra1, and Ra2. PPT-type ginsenosides include Re, Rf, Rg1, Rg1, Rg2, and Rh1 (Shin et al., 2015). Ginsenosides with a pentacyclic triterpene backbone, such as Ro, are classified as oleanolic acids (Nag et al., 2012). The last ginsenoside is the ocotillol type, which has a five-membered epoxide ring at C-20, and the rare ginsenoside P-F11, which is classified in it (Nakamura et al., 2007). Figure 6 and Table 1 show the characteristic ginsenosides with different substituent groups that are commonly observed in the basic chemistry of the four ginsenosides. The number and position of sugar molecules, hydroxyl portion of the dammarane backbone, and stereoisomeric position of C-20 have profound effects on the biological activity of ginsenosides (Cheong et al., 2015). Some studies have indicated that 20(R)-Rg3 exhibits greater antioxidative stress activity than 20(S)-Rg3, depending on the stereoisomeric position of C-20 (Li et al., 2014). More reassuringly, additional studies have identified that modified versions of some secondary metabolites of ginsenoside conversion or relevant drug carriers possess enhanced biological activity and exhibit unique pharmacological activity relative to the parent compound (Zheng et al., 2017).

**TABLE 1:** Various types of ginsenosides with different substitute groups.

Name	Formula	R1	R2
Protopanaxadiol ginsenosides			
Rb1	C_54_H_92_O_23_	Glc (2–1)Glc	Glc (6–1)Glc
Rb2	C_53_H_90_O_22_	Glc (2–1)Glc	Glc (2–1)Arap
Rb3	C_53_H_90_O_22_	Glc (2–1)Glc	Glc (2–1)Xyl
Rc	C_53_H_90_O_22_	Glc (2–1)Glc	Glc (2–1)Araf
Rd	C_48_H_82_O_18_	Glc (2–1)Glc	Glc
Rg3	C_42_H_72_O_13_	Glc (2–1)Glc	H
Protopanaxatriol ginsenosides			
Rg1	C_42_H_72_O_14_	Glc	Glc
Rg2	C_42_H_72_O_13_	Glc (2–1)Rha	H
Re	C_48_H_82_O_18_	Glc (2–1)Glc	Glc
Rf	C_42_H_72_O_14_	Glc	H
Oleanolic ginsenoside			
Ro	C_48_H_76_O_19_	GlcUA (2–1)glc	Glc
Ocotillol ginsenoside			
P-F11	C_42_H_72_O_14_	Glc (2–1)Rha	None

**FIGURE 7:**
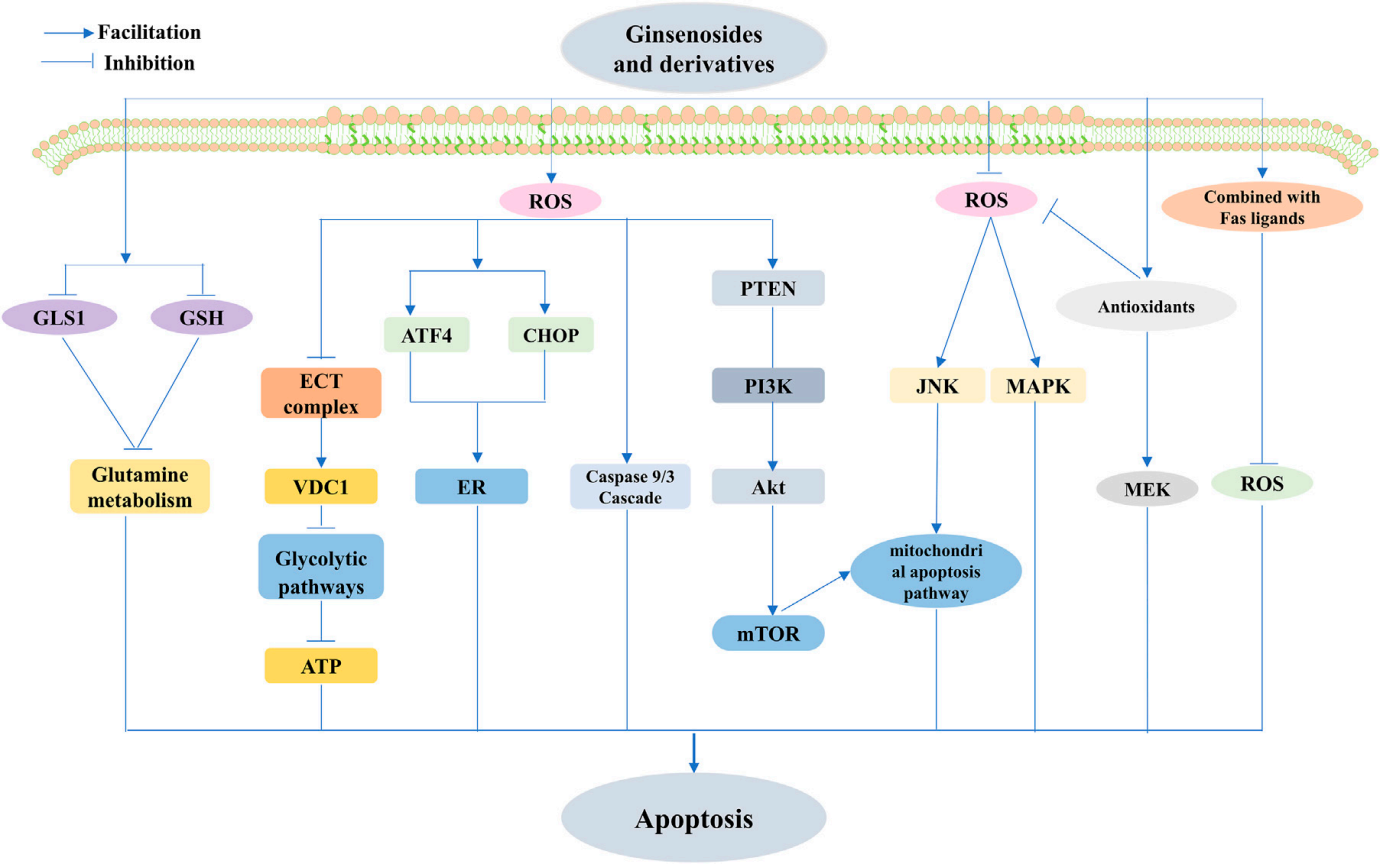
Ginsenosides modulate ROS-triggered apoptosis. Ginsenosides modulate ROS-triggered apoptosis involving mitochondria-associated, targeted endoplasmic reticulum (ER), and energy metabolism-mediated triggered apoptotic pathways. Ginsenosides promote apoptosis by increasing ROS production, and ginsenosides activate the caspase 9/3 cascade and mitochondria-mediated apoptotic pathways in cancer cells through ROS-dependent mechanisms and also induce mitochondria-dependent apoptosis by elevating the ROS-mediated PTEN/PI3K/Akt/mTOR signaling pathway. Increased ROS production also stimulates the upregulation of activating transcription factor-4 (ATF4) and C/Ebp-homologous protein (CHOP) The increased production of ROS also stimulates the upregulation of ATF4 and CHOP, as well as ER stress and inhibition of electron transport chain (ETC) complex formation, upregulation of voltage-dependent anion channel 1 (VDAC1), interference with the glycolytic pathway, reduction of glutaminase 1 (GLS1) and glutathione (GSH), and inhibition of glutamine metabolism to promote apoptosis; reduction of ROS induced by ginsenosides also mediates apoptosis, and reduced ROS-mediated upregulation of c-Jun N-terminal kinase (JNK) activation mediates the mitochondrial apoptosis pathway, and also, inhibition of antioxidant enzyme activity inhibits mitogen-activated extracellular signal-regulated kinase (MEK) and mitogen-activated protein kinase (MAPK). The combination of Rg3 and TNF-related apoptosis-inducing ligand (TRAIL) inhibited ROS to promote apoptosis.

**FIGURE 8:**
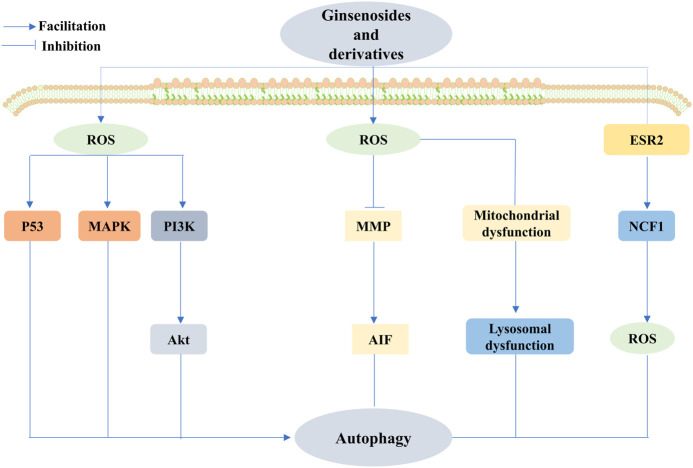
Ginsenosides promote or inhibit autophagy by regulating ROS to treat cancer. Ginsenosides can act as an autophagy inhibitor, mediating the production of ROS, inhibiting the mitochondrial membrane potential, and promoting the release of apoptosis-inducing factor (AIF) from mitochondria, thus causing nuclear translocation and promoting apoptosis; it can also increase ROS to impair mitochondrial function, leading to impaired lysosomal function and cell death. In addition, ginsenosides can also inhibit autophagy through the regulation of the estrogen receptor 2 (ESR2)-NCF1(neutrophil cytosolic factor 1)-ROS axis to exert anticancer effects. Conversely, ginsenosides exert anticancer effects through the regulation of ROS-mediated signaling pathways, including the upregulation of p53 signaling, and activation of mitogen-activated protein kinase (MAPK) and PI3K/Akt signaling pathways to promote autophagy to exert anticancer effects.

**FIGURE 9:**
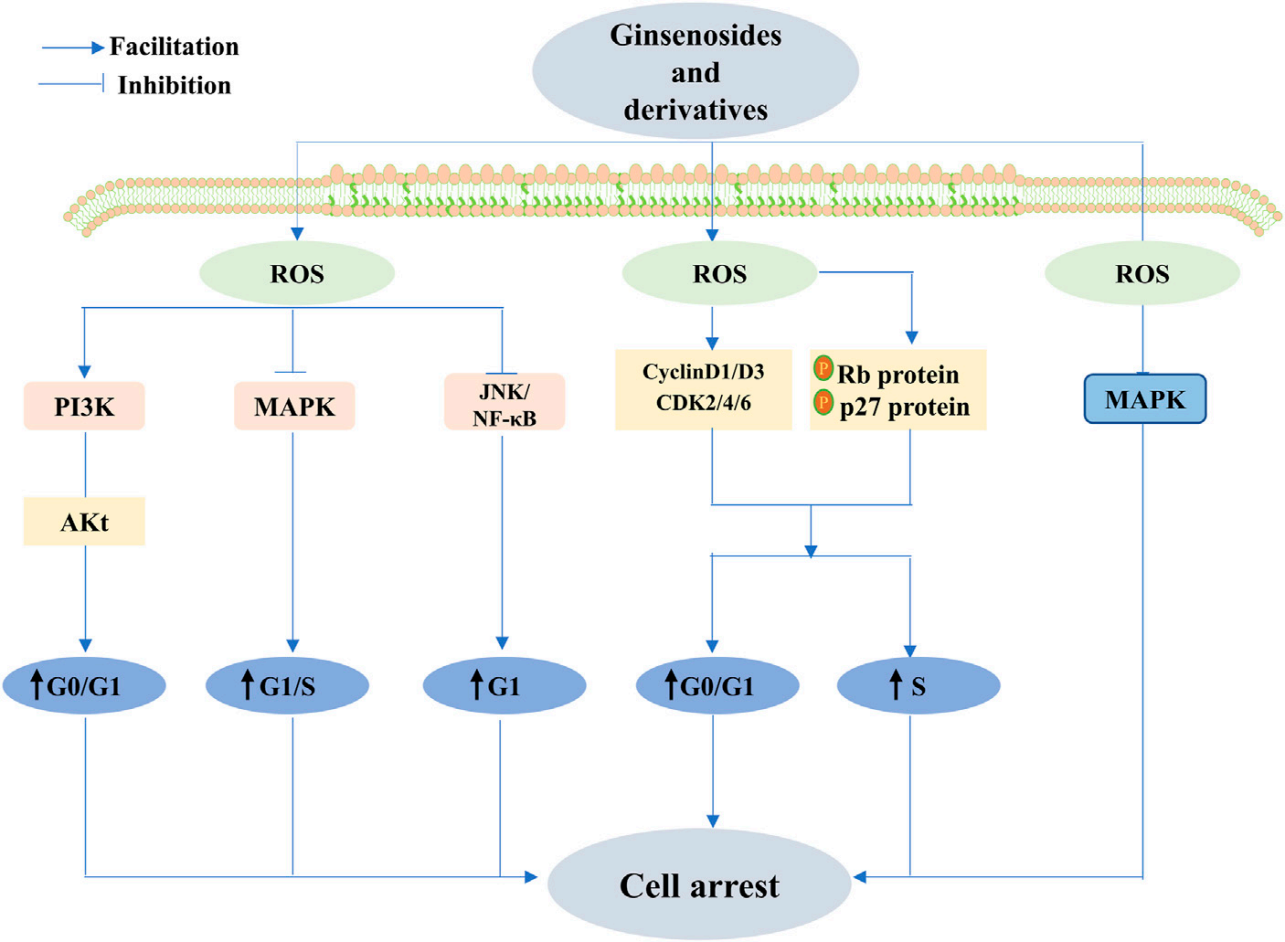
Ginsenoside exhibits anticancer activity through different stages of cell cycle arrest. Ginsenosides exert anticancer effects by reducing reactive oxygen species levels and activating MAPK. Conversely, ginsenosides activate the PI3K/Akt signaling pathway and c-Jun N-terminal kinase (JNK)/nuclear factor κB (NF-κB) and inhibit the MAPK signaling pathway by increasing ROS, causing cell cycle arrest in the G0/G1 phase, G1 phase, and G1/S phase, respectively, and promoting apoptosis. D1, cell cycle protein D3, cyclin-dependent kinase 2 (CDK2), cyclin-dependent kinase 4 (CDK4), and cyclin-dependent kinase 6 (CDK6), and a decrease in the expression of these proteins could promote the development of cell cycle arrest and anticancer effects.

**FIGURE 10:**
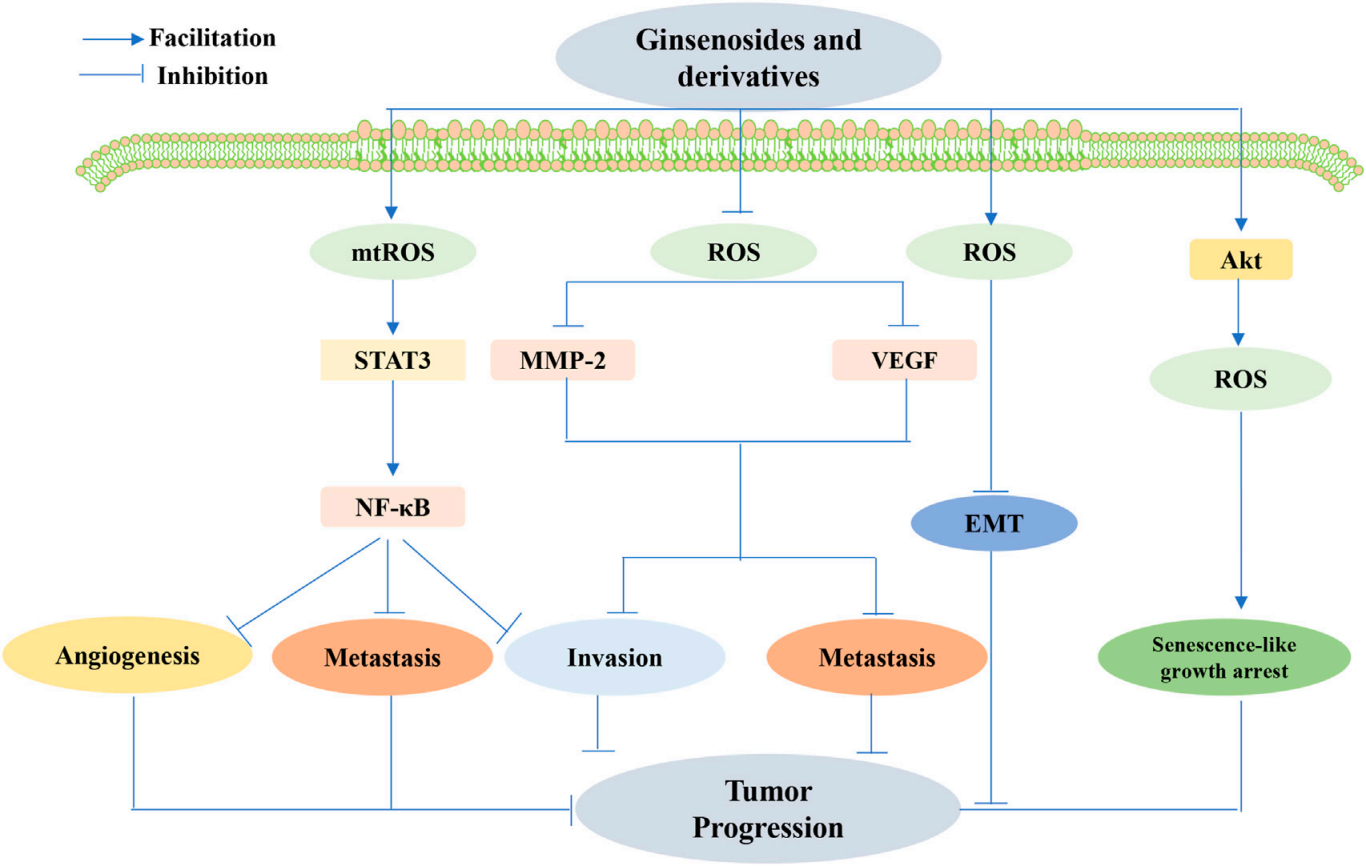
Ginsenoside-mediated interference with angiogenesis, metastasis, and invasion by ROS inhibited tumor development. Ginsenosides decreased ROS production, inhibited matrix metalloproteinases-2 (MMP-2) and VEGF expression, and suppressed tumor cell invasion and metastasis. Ginsenosides also promoted ROS produced in the mitochondria (MtROS)-mediated inhibition of the signal transducer and activator of the transcription 3(STAT3)/nuclear factor κB (NF-κB) signaling pathway, thereby inhibiting angiogenesis, metastasis, and invasion of cancer cells. Ginsenosides may also inhibit tumor progression by increasing ROS production and regulating the activation of epithelial–mesenchymal transition (EMT) conversion molecules. Targeted senescence may also be a promising approach for cancer treatment, where ginsenosides exert anticancer effects by inducing ROS production through the activation of Akt and senescence-like growth arrest.

To date, a comprehensive description of the functions of ginsenosides is available, among which their anticancer effect has been in full swing (Zhang et al., 2021). Concerning different cancers, ginsenosides act as anticancer agents by modulating different mechanisms, namely, mediation of apoptosis, proliferation, cell cycle arrest, metastasis, angiogenic effects, autophagy, reversal of multidrug resistance, and enhancement of chemotherapeutic effects, all of which are possible via the modulation of ROS (Figures 7–10). The following section focuses on the exploration of the mechanisms underlying the anticancer activity of different classes of ginsenosides against this target.

### 5.2 Studies on the regulation of ROS by the natural product ginsenoside to act as an anticancer agent

#### 5.2.1 Anticancer effects of ginsenosides through the regulation of ROS-mediated apoptosis

Unsuitable ROS levels reinforce cell death, and inverse signals for cell survival are heightened. The survival signal is mediated by the mitochondrial ROS activation of protein kinases and NF-κB, leading to the upregulation of antioxidant proteins (Cao et al., 2020). Apoptosis triggered by natural ginsenosides via the modification of ROS, involving but not limited to endogenous and exogenous apoptotic pathways, tends to come into vogue in the treatment of cancer. In addition, many researchers have emphasized the superior role of ROS in targeting ER- or energy metabolism-mediated pathways that trigger apoptosis in various cancer cell lines. Natural ginsenoside extracts that modulate the role of ROS (lower or higher) in the aforementioned apoptotic pathways in cancer therapy should not be underestimated (Kim et al., 2017; Seervi et al., 2018; Cao et al., 2020; Zheng et al., 2020).

It was discovered that ultrasound-treated ginseng berry extract (UGBE), containing multiple ginsenosides, including Rh1, Rg2, and ginsenosides Rg3 and Rh2, could activate the mitochondria-mediated apoptotic pathway in cancer cells through an ROS-dependent mechanism. The same conclusion was obtained in ginsenoside Rh2- and Rg3-treated human leukemia Jurkat cells (Park et al., 2012; Chen et al., 2015; Jung et al., 2016; Xia et al., 2017). Consistent with previous studies, Rg3 enhanced the level of ROS and led to the caspase 9/3 cascade, yet its induction of apoptosis was not significantly disturbed by the application of NAC, suggesting that the increased ROS associated with Rg3 was not the culprit of apoptosis (Kim B.-M. et al., 2013). The underlying mechanism behind this process still requires further exploration. Several studies have uncovered a specific mechanism by which Rk1 induces mitochondria-dependent apoptosis by mediating the PTEN/PI3K/Akt/mTOR signaling pathway via elevated ROS levels in MCF-7 cells (Hong and Fan, 2019b). However, ginsenoside-induced reduction of ROS, leading to the activation of signaling pathways, is also an effective treatment for the induction of apoptosis (Huynh et al., 2021), which is consistent with the finding that diminished ROS-mediated upregulation of JNK activation plays an integral role in the ginsenoside-mediated mitochondrial apoptosis pathway (Ham et al., 2006; Mao et al., 2014). However, Chu et al. demonstrated that Rg1 exerts cytoprotective effects in MPP + -treated SHSY5 cells by reducing the ROS-mediated regulation of JNK (Chu et al., 2020).

Ginsenoside-mediated dysregulation of antioxidant enzyme activity is associated with endogenous apoptosis. Choi et al. identified that ginsenoside Rg3 regulates CAT activity and inhibits the MEK signaling pathway to mediate apoptosis (Choi et al., 2013). Rg5 and Rk1 mediate apoptosis in lung cancer cells through the regulation of GSH (Kwak and Pyo, 2016). Furthermore, Lu et al. found that Rk1 could promote apoptosis in cervical cancer cells by reducing GLS1 and GSH and inhibiting glutamine metabolism (Lu et al., 2022). However, one study pointed out that Rg1 could inhibit DMBA-mediated carcinogenesis by restoring antioxidant enzyme activity (Chu et al., 2020). FBG has cytoprotective functions by suppressing MAPK and lowering ROS levels caused by the induction of antioxidant enzyme activity (Bak et al., 2014). However, Rh2, by enhancing ROS, partially counteracts p53-induced apoptosis by activating AMPK and NF-κB signaling pathways to promote cancer survival and growth and can exert better anticancer effects when applied in combination with antioxidants (Li et al., 2011).

Rh2 and compound K evoke apoptosis in a cystathionine- and p38 MAPK-dependent manner in astrocytoma cells, and combined treatment with Fas ligands exerts synergistic cytotoxic effects by reducing intracellular ROS (Choi and Choi, 2009). Lee et al. found that the combination of Rg3 and TRAIL resulted in a noticeable improvement in ROS production to either one alone. Although the antioxidant NAC effectively inhibited ROS production, it failed to suppress apoptotic cell death induced by Rg3 combined with TRAIL, suggesting that ROS may not be required for Rg3 sensitivity to TRAIL (Lee et al., 2013).

ER stress-mediated apoptosis cannot be minimized by ginsenoside treatment. Ginsenoside Rh2 has been shown to facilitate apoptosis in lung cancer cells, especially through the induction of ROS production, stimulating the upregulation of ATF4 and CHOP, with ER stress further inhibiting cell proliferation (Ge et al., 2017). Similarly, Wu K. et al. found that ATF4 knockdown attenuated the proapoptotic effect of Rg3 in gallbladder cancer cell lines by inhibiting ROS (Wu K. et al., 2018). As such, it has been suggested that Rh2 enhances ROS, inhibits ETC complex formation, affects energy stress, upregulates VDAC1, and interferes with glycolytic pathways to promote apoptosis (Liu et al., 2020; Liu et al., 2021).

#### 5.2.2 Anticancer effects of ginsenosides through the regulation of ROS-mediated autophagy

The dual role of autophagy in cancer progression and inhibition remains controversial. Natural ginsenosides can promote or inhibit autophagy by regulating ROS for cancer therapeutic effects.

It has become more unambiguous that Rg3, as an inhibitor of autophagy, can mediate the production of ROS, decrease the mitochondrial membrane potential, and consequently induce apoptosis, all of which induce the release of AIF from the mitochondria and enable nuclear translocation that leads to HeLa cancer cell death (Bian et al., 2019). G-Rh2 showed a significantly stronger inhibition of autophagy than Rg3. The specific mechanism is to impair mitochondrial function through increased ROS, leading to defective/impaired lysosomal acidification/function and, ultimately, HGC-27 cell death (Han et al., 2020). The conclusion that Rh2 inhibits autophagy in cervical cancer cells was also drawn by Wang et al. in cervical cancer cells (Wang J. et al., 2020). Furthermore, by the preapplication of N-acetyl-l-cysteine (NAC) to esophageal cancer cells, Bian et al. identified that ginsenoside Ro-induced elevated expression of autophagy-associated proteins, such as LC3B-II, was significantly reversed, precisely through the modulation of the ESR2-NCF1-ROS axis. Importantly, the study also observed that Ro enhanced the cytotoxicity of 5-fluorouracil (5-Fu) by delaying CHEK1 (checkpoint kinase 1) degradation and downregulating the DNA replication process, which strongly suggests that G-Ro can be used as an effective anticancer agent to overcome chemoresistance in combination therapy (Zheng et al., 2016). However, growing evidence suggests that the promotion of autophagy plays a critical role in cancer treatment. Hwang et al. demonstrated that the activation of mitochondrial autophagy is essential for combating lung cancer in Rg3-enriched red ginseng, which is initiated by the increase in ROS (Hwang et al., 2022). Likewise, several native ginsenosides exert anticancer effects through the ROS-mediated regulation of signaling pathways. Rh4 mediates ROS production to induce the upregulation of p53 signaling, thereby activating autophagy and positively regulating iron death to promote colorectal cancer cell death (Wu et al., 2022). However, Wu et al. revealed that Rh4 induces autophagy by facilitating the ROS/JNK/P53 pathway, and interestingly, the upregulation of autophagy-associated proteins ATG7 and Beclin-1 is involved in the regulation of autophagy (Wu Q. et al., 2018). Rg5 and Rh1 promote ROS production, and trigger MAPK activation and the PI3K/Akt signaling pathway, respectively, to promote autophagy and cell cycle arrest, inhibit cancer cell proliferation, and promote the onset of apoptosis (Liu and Fan, 2019).

#### 5.2.3 Anticancer effects of ginsenosides through the regulation of ROS-mediated cell cycle arrest

Numerous studies have suggested that natural ginsenoside products display pronounced anticancer activity at different stages of cell cycle arrest. Lu et al. found that ginsenoside Rk1 effectively arrests the cell cycle in the S-phase and induces apoptosis in breast cancer MCF-7 cells. Moreover, increased p53 and p21 proteins and the downregulation of cyclin A and CDK2 might contribute to the anticancer effects of Rk1. Rk1 may regulate the proteins via the upregulation of the ROS-mediated PTEN/PI3K/Akt/mTOR signaling pathway, sequentially inhibiting proliferation and apoptosis (Lu et al., 2022). Hong et al. observed identical alterations in ROS and proteins in MDA-MB-231 triple-negative breast cancer cells, with the difference being that the cells blocked the cell cycle in the G0/G1 phase (Hong and Fan, 2019a). Huynh et al. revealed that the accumulation of the G0/G1 phase in cancer cells incubated with Rh1 was enhanced, along with marked improvement in phosphorylated Rb protein levels and p27 protein expression; meanwhile, the expression of cycle-related proteins—including cyclin D1, cyclin D3, CDK2, CDK4, and CDK6—diminished. Moreover, the mentioned reaction coincided with the ROS-mediated PI3K pathway (Huynh et al., 2021), and these results are similar to the findings of Jin et al. (2022). A decrease in ROS levels exerts an aggressive effect on cell cycle arrest. Shui et al. showed that Rg3 has promising anticancer activity against lung cancer by decreasing reactive oxygen levels, activating cell cycle-associated proteins, and controlling MAPK relevant to proliferation (Sun et al., 2016). Conversely, Jeon et al. applied NAC to counteract the G1-S phase arrest due to Rg2 via the activation of AMPK and the regulation of cell cycle regulators induced by increased ROS, implying that increased ROS can also exhibit excellent anticancer effects by promoting cell cycle arrest (Jeon et al., 2021). Comparably, Rg1 exerts apparent cancer-fighting actions on paclitaxel-resistant nasopharyngeal carcinoma cells by upregulating ROS to block the PI3k/Akt signaling pathway (Li et al., 2019). Rg18, in a similar manner, or at least in part, downregulated the JNK/NF-κB signaling pathway to lead to G1-phase cell arrest to curb cancer cell proliferation of A549 cells to combat cancer (Leem et al., 2018). It has, therefore, been abundantly demonstrated that ginsenosides can exhibit exceptional therapeutic anticancer abilities at different stages of the cell cycle via the modulation of ROS.

#### 5.2.4 Anticancer effects of ginsenosides through the regulation of ROS to inhibit angiogenesis, invasion, and other mechanisms

Targeting cancer metastasis by interfering with angiogenesis and reducing invasiveness is an important aspect of ginsenoside therapy. Previous research revealed that G-Rh2 shows both high antioxidant activity and low toxicity by decreasing the production of ROS and subsequently repressing MMP-2 and VEGF expression, thereby suppressing the invasion and metastasis of oral squamous cell carcinoma cells (Ping et al., 2020). Nonetheless, Jin et al. concluded that Rh1 repressed cancer cell angiogenesis, metastasis, and invasion by boosting MtROS-mediated inhibition of the STAT3/NF-KB signaling pathway (Jin et al., 2021). Additionally, the ginsenoside Rg1 may suppress the progression of DMBA-induced breast cancer via a concentration-dependent increase in ROS production and by modulating the activation of molecules involved in cell proliferation, apoptosis, invasion, angiogenesis, and EMT conversion (Chu et al., 2020). Targeting senescence is also a more promising approach to treat cancers, as suggested by Sin S et al., who delivered chronic treatment with sub-apoptotic concentrations of 20(S)-Rg3 to induce ROS production via Akt activation and p53/p21-dependent senescence-like growth arrest in glioma cells (Sin et al., 2012). This study provides valuable insights into the future development of 20(S)Rg3 as a novel anticancer agent.

### 5.3 Studies on the regulation of ROS by ginsenoside derivatives, nanoparticles, and other ginsenosides

This section highlights the different classes of ginsenoside derivatives, nanomaterial carriers, and other ginsenosides that mediate apoptosis, cell cycle arrest, and autophagy through direct or indirect modulation of ROS to exert anticancer effects. Studies have found that ginsenoside metabolite K (GCK) exhibits anticancer activity by boosting ROS-mediated alteration of the mitochondrial membrane potential in neuroblastoma or cervical cancer cells, functioning as an autophagy inhibitor or modulating caspase-3/9 and PARP intrinsic apoptotic pathways (Oh et al., 2019; Yin et al., 2021). Identically, Gao H. et al. and Wang X.D. et al. discovered that 2-deoxy-Rh2, a novel 20(S)-Rh2 derivative with enhanced anticancer activity, was designed and synthesized by the hybridization of protopanaxadiol and 2-deoxyglucose (2-DG), and 2-pyrazine-PPD, a derivative obtained by introducing a pyrazine ring into 25-OH-PPD, also mediated ROS-induced mitochondrial dysfunction to cause apoptosis in cancer cells. Dissimilarly, it is noted that the former also enhances the proapoptotic effect by affecting glycolysis and decreasing increased ATP (Gao et al., 2020), while the latter reinforces the antiapoptotic effect by affecting PERK/EIF2A/ATF4 and CHOP expression to affect ER stress, thus reinforcing the pro-cancer effect (Wang X. D. et al., 2020). An alternative derivative of PPD, 12-chloracetyl-PPD, a 25-OH-PPD analog synthesized by the addition of C-12-OH to chloroacetyl, can contribute to the counteracting effect by causing G2/M-phase cell cycle arrest via increased ROS levels, thus downregulating MDM2 and upregulating P53 (Wang et al., 2017). In addition, ROS-mediated signaling pathway activation positively contributes to anticancer effects. CK functions in colon and bladder cancers by mediating the activation of JNK and P38MAPK (Kim A. D. et al., 2013; Wang et al., 2013). AD-1, extracted from ginseng berries, can also promote apoptosis in lung cancer cells by mediating the activation of p38mapk, which has been validated using NAC (Zhang et al., 2013). 1c, a ginseng saponin derivative, functions as an anticancer agent through increased ROS-mediated inhibition of the Wnt/β-catenin signaling pathway (Wang et al., 2018).

The buildup of ginsenoside nanoparticles and related vectors has been shown in comparison to ginsenosides, which evoke stronger cytotoxicity by triggering a somewhat higher production of ROS, thereby inducing stronger cytotoxicity but lower toxicity to normal cells. Rh2HAZNO nanoparticles, a hyaluronic acid (HA)-functionalized zinc oxide (ZnO) nanocomposite (HA-ZnONcs) prepared using the coprecipitation method, were further functionalized with ginsenoside Rh2 through a cleavable ester bond of carbodiimide chemistry, generating ROS to induce apoptosis in cancer cells through the activation of the caspase-9/p38mapk signaling pathway (Kim et al., 2019). Xu et al. (2020) applied larulan polysaccharides grafted with allantoic acid and -lipoic acid (-LA) to obtain a pH and redox dual-responsive copolymer, LA-conjugated N-larulan allantoic acid (LA-URPA), enabling the copolymer LA-URPA-encapsulated ginsenoside Rh2 to form Rh2 nanoparticles (Rh2 NPs) that exert stronger proapoptotic effects by increasing ROS and downregulating antioxidant enzymes, such as SOD, CAT, and GSH (Xu et al., 2020). Moreover, the buildup of ginsenoside nanoparticles can reduce the side effects caused by ginsenosides. A previous study reported that DOX@Rg1 nanoparticles could attenuate free DOX-induced ROS generation and relevant apoptosis in H9C2 cells and mitigate cardiotoxicity; however, their toxicity was significantly increased in cancer cells (Li et al., 2021). Thus, constructing ginsenoside carriers can be a prospective therapeutic approach to enhance anticancer efficiency and reduce associated toxic side effects.

### 5.4 Study of ginsenosides as adjuvant drugs to promote anticancer drug sensitivity

Despite studies supporting the therapeutic benefits of ginsenosides in combination with chemotherapeutic agents, their specific mechanisms have not been fully elucidated. A worthy case of back-citation is ginsenoside Ro, a novel autophagy inhibitor, that activates estrogen receptor 2 (ESR2), which consequentially activates a subunit of NADPH oxidase termed NCF1/p47PHOX (neutrophil lysyl factor 1); this cascade ultimately leads to 5-fluorouracil (5-Fu)-induced chemoresistant esophageal cancer cell death by ROS production and marked inhibition of autophagic fluxes (Zheng et al., 2016). Similarly, Chen et al. determined that Rh2, via regulatory autophagy, strengthened both NSCLC A549 and H1299 apoptosis caused by cisplatin, mostly through the induction of PD-L1 expression via the ROS-EGFR-PI3K-AKT-autophagy pathway. Interestingly, a significant increase in SOD activity was detected after Rh2 administration, whereas no significant change in GSH was noted, and the side effects of hearing loss due to ROS production were significantly attenuated (Chen et al., 2020). The upregulation of the Nrf2-driven antioxidative signaling pathway may also contribute to chemoresistance in cancer cells. Chian et al. studied this mechanism and verified that the ginsenoside Rd could, indeed, work against cisplatin-resistant lung cancer by downregulating this signaling pathway (Chian et al., 2019), which is consistent with the mechanism derived by Popov et al. in a study on the enhancement of the anticancer effect of DOX by RH2 (Popov et al., 2022). Moreover, both Rg1 and Rh2 may serve as chemosensitizers of doxorubicin, which suppresses the NF-κB signaling pathway to inhibit Dox-induced SASP (IL-8 and TNFα), thereby rescuing the viability of normal mammary epithelial cells and maintaining an inhibitory effect on cancer proliferation. Critically, such regulation is correlated with decreased ROS elicited by a significant increase in Rh2-mediated SOD1 and SOD2 and the regulators SIRT3 and SIRT5 at the protein level (Hou et al., 2020). However, when Rh2 was combined with DOX to treat breast cancer cells, the significantly higher ROS levels not only altered MMP, leading to cytochrome C release, but also acted as a synergistic cytotoxic agent by breaking single- and double-stranded DNA, thus activating apoptotic signaling (Liu et al., 2022). In addition, there is evidence of ginsenosides weakening the aggressiveness of antineoplastic drugs. Rg3 abrogated gemcitabine (GEM)-induced production of ROS-mediated activation of the Akt and extracellular signal-regulated kinase (ERK) pathways; moreover, it suppressed nuclear accumulation of NF-κB and HIF-1α to decrease PTX3, with significance in GEM-induced drug resistance, thereby dampening the GEM-induced aggressiveness of lung cancer cells (Ahmmed et al., 2019).

### 5.5 Limitations

Taken together, there is an encouraging anticancer effect of ginsenosides in both cellular and animal models. However, preclinical studies on ginsenosides are scarce and the corresponding evidence is insufficient, possibly due to the following reasons: 1) a large molecular weight is composed of a large number of glycosides and needs to be metabolized into rare ginsenosides to have higher pharmacological activity *in vivo*. However, the low content of rare ginsenosides and the difficulty of isolation have limited the clinical application of ginsenosides (Fan et al., 2020; Hou et al., 2021); 2) due to poor absorption and bioavailability of ginsenosides, their anticancer effects are significantly reduced and species specificity render ginsenosides less dose-referenced for *in vitro* cellular and animal experiments. Using a monolayer model of human intestinal Caco-2 cells, Liu et al. demonstrated that G-Ra3 is poorly absorbed in the intestines (Liu et al., 2009) and that G-Rg1/Rb1 can achieve similar results, which may be related to the low permeability of the intestinal mucosa and first-pass action of the liver (Han and Fang, 2006; Kim, 2013). Encouragingly, given the aforementioned limitations of ginsenosides, studies on modified methods are emerging, investigating chemical modifications that alter the ginsenoside backbone structure (Ma et al., 2020) and the development of nano-delivery systems (Xu et al., 2020); ultimately, such modifications may effectively enhance pharmacological activity and bioavailability, as well as the targeting of ginsenosides to better treat cancer. It is reasonable to believe that with continuous technological development in pharmaceuticals and biochemistry fields, additional methods will be developed to improve the bioavailability of ginsenosides at a low economic cost, which is of great significance to fully utilize ginseng resources for the benefit of the public.

## 6 Conclusion

Given the growing body of literature, ginseng shows significant potential in cancer treatment. Ginsenosides exert their anticancer effects by modulating the majority of well-known carcinogenic modulators. The adjusted redox state of cancer cells can be used to design promising therapeutic strategies. ROS play a bidirectional role in cancer, which depends on the genetic background of the cancer, type of ROS involved, and level and duration of ROS exposure. Ginsenosides and their related derivatives exert superior anticancer effects by reacting with different cellular signaling cascades to directly or indirectly modulate ROS and impair redox homeostasis in cancer cells. Encouragingly, in the present cellular, animal, or preclinical studies involving ginsenosides, no cancer-promoting effects have been found, in the context of the possible mechanisms involved in the modulation of immunity and suppression of inflammatory responses besides those mentioned previously (Wong et al., 2015). In addition, since ginsenosides are known to lead to a variety of cell death processes, it is rare for cancer cells to be resistant to their induced cell death. Remarkably, ginsenosides can selectively kill tumor cells with relatively little toxicity to normal cells. Such selective toxicity and the optimization of this selection could be a valuable area of concern for future research and exploration.
